# Characterization of the endophytic bacterial community of *Bituminaria bituminosa* plant grown *in vitro* and its interaction with the plant extract

**DOI:** 10.3389/fpls.2022.1076573

**Published:** 2023-01-18

**Authors:** Carolina Chiellini, Marinella De Leo, Vincenzo Longo, Ylenia Pieracci, Laura Pistelli

**Affiliations:** ^1^ Institute of Agricultural Biology and Biotechnology, Italian National Research Council, Pisa, Italy; ^2^ Department of Pharmacy, University of Pisa, Pisa, Italy; ^3^ Interdepartmental Center for Instrument Sharing of Pisa University, Pisa, Italy; ^4^ Department of Agriculture, Food and Environment, University of Pisa, Pisa, Italy

**Keywords:** *in vitro* cultures, endophytes, indole 3-acetic acid, antagonism, UHPLC-MS, pterocarpans, furanocoumarins, prenylated flavonoids

## Abstract

**Introduction:**

*Bituminaria bituminosa* is a medicinal plant recognized for its phytochemicals, such as furanocoumarins, pterocarpans, and flavonoids. Since the secondary metabolism is influenced by the plant-endophyte interactions, the endophytic bacterial community of *B. bituminosa* was explored and the possible interactions with the plant were described.

**Materials and methods:**

Different bacterial strains were isolated from different organs of *in vitro* plants as shoots, roots, and seeds. The bacterial strains were identified and phenotypically characterized for different traits; strains were also exposed to different concentrations of *B. bituminosa* plant extract showing different susceptibility, probably determined by different secondary metabolites produced by the plant in the different organs (i.e. aerial parts and roots).

**Results and discussion:**

Bacterial strains showed different phenotypic characteristics; the 6 detected haplotypes were dominated by a single species related to *Stenotrophomonas rhizophila*. Endophytes isolated from the aerial parts produced a higher indole-3-acetic acid (IAA) amount than those of the roots, while all strains were unable to produce biosurfactants and antagonistic activity toward the other strains. The research opens new perspectives for future analysis addressed to test the susceptibility of the endophytic bacterial community of *B. bituminosa* toward the pure compounds extracted from the plants, and to investigate the role of these compounds on the distribution of endophytes within the different plant tissues.

## 1 Introduction

Endophytes are fungal and bacterial microorganisms inhabiting the inner tissues of plants, whose importance for the host plant species has been largely investigated for decades (Reinhold-Hurek and Hurek, 2011). Endophytic bacterial strains might help the plant in its development by producing secondary metabolites with growth-promoting (Plant Growth Promotion, PGP) activity (Santoyo et al., 2016). Indole-3-acetic acid (IAA), is a molecule of bacterial origin considered important for plant-growth promotion. It is one of the most important phytohormones, also playing a role in alleviating different biotic and abiotic stresses in the plant species (Shahzad et al., 2017). IAA production varies among bacterial species and strains (Rashid et al., 2012). Other than PGP traits, the plant endophytic community can have a role in the plant defense inducing secondary metabolite production with an antimicrobial effect, which might be useful to the plant in the defense against other putative phytopathogenic microorganisms (Dini-Andreote, 2020; Mengistu, 2020). Finally, the plant endophytic community might have a role in the plant adaptation to hostile environments, such as soil enriched with metals (Ma et al., 2016) or hydrocarbons (Marchut-Mikolajczyk et al., 2018), producing molecules – i.e. biosurfactants, siderophores, etc. - that “decontaminate” the soil microenvironment around the plant rhizosphere, helping plants to adapt to biotic and abiotic stress conditions of their habitat. On the other hand the plant itself might offer the endophytic microorganisms a safe and protected environment to grow in, as well as nutrient availability (Bacon and Hinton, 2006).

Recent studies evidenced a kind of plant-endophytic community communication by demonstrating that the plant might induce a kind of compartmentalization among the different plant organs (Rossmann et al., 2017). In particular, antagonistic interactions among the strains seem to play a role in shaping the endophytic community within the plant species (Maida et al., 2016), together with other bacterial phenotypic characteristics (Mengoni et al., 2014; Maggini et al., 2018). Interestingly, *in vitro* studies suggested that endophytic bacterial strains might contribute to the therapeutic properties of medicinal plants since the plant’s secondary metabolism is affected by the plant-endophyte interactions (Maggini et al., 2017). Accordingly, endophytic microorganisms can produce high-value bioactive molecules, and contribute to the medicinal properties of the plants (Venugopalan and Srivastava, 2015). Moreover, endophytic microorganisms may produce secondary metabolites promoting plant growth, affecting the uptake or redistribution of resources which, in turn, can improve the health of the host plant and consequently the accumulation of the bioactive metabolites (Ye et al., 2021). Finally, recent advances in the study of endophytic communities reveal that bacterial endophytes can produce molecules with antibacterial activity against human pathogenic bacteria (Chiellini et al., 2017; Presta et al., 2017), opening new perspectives for further investigations aimed at characterizing the bioactive molecules produced by endophytes, to evaluate their possible application for biotechnological purposes, with a particular interest in the therapeutic field.

*Bituminaria bituminosa* (L.) C.H. Stirt. (syn. *Psoralea bituminosa* L.), belonging to the Fabaceae family is a xerophytic shrub widely distributed in the coastal Mediterranean area, with a potential role in the protection of coastal soil from erosion (Andreu et al., 1995); the plant is commonly used as hay and forage for herbivores, mainly goats (Sternberg et al., 2006). Another typical role is the phytostabilization of former mining zone contaminated by heavy metals (Martínez-Fernández et al., 2011; Pistelli et al., 2017). *B. bituminosa* is recognized for its peculiar foliar smell of bitumen, due to the combination of phenolics, sulphurated compounds, sesquiterpenes, and probably short-chain hydrocarbons (Tava et al., 2007). The plant is known as a source of several phytochemicals, mainly furanocoumarins, pterocarpans, and flavonoids. The furanocoumarins are phytoalexins, synthesized against fungal infection and insects. Psoralen and its angular form angelicin are the furanocoumarins found in several organs of *B. bituminosa* wild plants and *in vitro* culture (Innocenti et al., 1997; D’Angiolillo et al., 2014; Pistelli et al., 2017). Psoralen is used in dermatology to treat human skin diseases, while angelicin shows calmative, sedative, and anticonvulsant activities. Erybraedin C and bitucarpin A are the main pterocarpans detected for the first time in *B. bituminosa* aerial parts (Pistelli et al., 2003). These metabolites show anti-inflammatory, antiviral, antiproliferative, and apoptotic (anti-tumor) activities (Maurich et al., 2006; Noccioli et al., 2014). Other secondary metabolites detected in *B. bituminosa* are flavonoids, documented for their antibacterial activity (Azzouzi et al., 2014; Ramli et al., 2022).

Former studies highlighted the diversity of secondary metabolite content in several organs, both in wild plants and *in vitro* organs such as calli, hairy roots, shoots, and roots (D’Angiolillo et al., 2014; D’Angiolillo et al., 2017). The *in vitro* culture demonstrated different production of metabolites: callus cultures and hairy roots produced mainly the isoflavone daidzein, and young roots furanocoumarins, while *in vitro* shoots showed the same production of adult plants, even though at very low concentration (D’Angiolillo et al., 2014; D’Angiolillo et al., 2017; Pistelli et al., 2017). Young plants inoculated with Arbuscular Mycorrhizal Fungi (AMF) showed the presence of all the above-mentioned secondary metabolites, although their concentrations were different between the vegetative and reproductive phases (Pistelli et al., 2017). The influence of AMF led to investigate if also endophytic bacteria could play a role in the production or activity of secondary metabolites.

In this work, the endophytic community of *B. bituminosa* plants grown *in vitro* in sterile conditions for 40 days was explored for the first time. Both molecular and phenotypic characterization of the strains isolated from roots, aerial parts, and seeds were performed. The stress resistance of the bacterial strains toward different conditions (i.e., salinity, water, antibiotics, and oxidative stress) was evaluated as well, together with the antagonistic activity among isolates. Finally, the growth of the bacterial strains in presence of the *B. bituminosa* wild plant extracts was evaluated to verify their eventual susceptibility. Overall, the bacterial community characterization of *B. bituminosa* and the possible interactions with the plant were explored and described.

## 2 Materials and methods

### 2.1 Chemicals

Analytical grade chloroform used for the extract preparation was purchased from Merck (Darmstadt, Germany), while UHPLC grade methanol, formic acid, and water were supplied from Romil-Deltek (Pozzuoli, Italy).

### 2.2 Plant materials

*Bituminaria bituminosa* (L.) C.H. Stirt. aerial parts were collected from the field in Elba Island in April 2022. Mature seeds were collected during the summer 2021. A voucher specimen was authenticated by S. Maccioni (Department of Biology of the University of Pisa) and deposited at the Botanical Garden of the University of Pisa (HHP-new acquisitions 3703/9).

### 2.3 Seeds germination and shoot growth

Seeds were sterilized as already published (D’Angiolillo et al., 2014) and transferred for germination and growth in full strength salts and vitamins Murashige and Skoog medium (MS0, Murashige and Skoog, 1962), containing 3% sucrose (w/v), agar 0.8%, plant preservative mixture (PPM) 0.05%, and adjusted to pH 5.8 before autoclaving. The explants were maintained in Magenta vessels at 22 ± 1°C, under 16/8 photoperiod conditions at the irradiance 50 μmol/m^2^ s (cool white fluorescent tubes, Phillips, Holland), until use (40 days).

### 2.4 Isolation of bacterial endophytes from seeds

Bacterial endophytes were first isolated from the seeds of *B. bituminosa*. Briefly, seeds were kept in concentrated H_2_SO_4_ for 50 min to remove external coats, and washed 3 times with distilled water. The sterilization proceeded with a solution 20% (v/v) of commercial sodium hypochlorite (final active Chlorite concentration 1%) for 15 min, washed 3-5 times with sterilized water. The seeds were kept in water in the dark for 2 days at 25°C to permit germination.

Fifteen seeds were disrupted in a sterile mortar with a 10 mL saline solution previously autoclaved (0.9% NaCl). Serial dilutions of the homogenized seeds were performed in a saline solution; 100 µL of each dilution (1:10, 1:100, and not diluted) were plated in triplicate in Tryptone Soy Agar (TSA) plates. The water obtained from the last washing in the seed sterilization protocol was plated as well, to evaluate the sterility and to check the absence of any microbial growth. Plates were monitored over 2 weeks; a single bacterial colony was grown and isolated in TSA medium as further described.

### 2.5 Isolation of bacterial strains from plants grown *in vitro* and ARDRA screening for haplotype attribution

After 40 days of *in vitro* growth, the absence of contamination was observed in all the magenta vessel tests. Ten plants were picked up in sterile conditions and for each one, the roots were separated from the aerial parts with a sterile scalpel. Roots and aerial parts were treated separately as a pool for the 10 plants. Each pool was homogenized in a sterile mortar with sterile saline solution and serial dilutions were performed. 100 µL of each dilution (1:10, 1:100, and not diluted) were plated in duplicate in TSA plates and monitored over 4 days. Bacterial plate counts were performed after 48 and 96 h and reported as results after 96 h in this work.

After 96 h, 21 isolated colonies were randomly chosen from the aerial parts; 21 isolates were recovered from the plates in which roots were plated.

A total of 43 isolates (21 from aerial parts, 21 from roots, and 1 from seeds) were analysed through ARDRA screening, following the protocol described in Gabriele et al (2022). Fragments showing an identical electrophoresis pattern were grouped in the same haplotype. According to Chiellini et al. (2018), Chao-1, Shannon, and Evenness diversity indices were calculated on the haplotypes obtained for each plant tissue using PAST3 software (HammerDavid A.T. and Ryan, 2001)

### 2.6 Molecular characterization of the isolated strains and phylogenetic analysis

Once the haplotype attribution was completed, one strain for each haplotype was chosen for the molecular characterization through 16S rRNA amplification and sequencing. The DNA was obtained from each bacterial strain through thermal lysis and the 16S amplification was conducted in the same conditions described in Gabriele et al (2022). The obtained amplicons were purified through ethanol precipitation and sent to Mycrosynth company (Germany) for sequencing. The obtained sequences were processed as described in Gabriele et al (2022). The phylogenetic analysis was conducted with the Maximum Likelihood method on a total of 77 sequences (71 high-quality sequences selected from international databases and 6 sequences belonging to our endophytic isolates).

### 2.7 Cross streaking, biosurfactant production, and IAA production assay in isolated endophytes

All the isolated bacterial endophytes were analysed for their ability in inhibiting the growth of each other. In particular, the ability of root endophytes was tested against the ones isolated from the aerial parts, according to the cross streak method described in Chiellini et al (2019), using the TSA agar medium and incubating the plates for 48 h at 22°C. A bacterial growth comparable to that of the control test (target strains without tester strains) was indicated as “+”; a slightly lower growth of target strains with respect to the control test was indicated as “+-”, a low growth respect to control test was indicated as “+–” while the total absence of growth was indicated as “-”.

The ability of all the isolated strains in producing biosurfactants was assessed through the Mineral Salts Agar medium method, as described in Siegmund and Wagner (1991), incubating the plates for 1 week at 22°C.

The IAA production was assessed in the liquid medium, according to the protocol of the estimation by the Salkowski reagent, with a colorimetric assay and a spectrophotometric quantification, as described in Gabriele et al. (2022). The quantification of the produced IAA was calculated through the construction of a standard curve using IAA at concentrations of 0, 1, 2, 5, and 10 µg/mL, diluted in the culture medium of the bacterial strains. The quantification of IAA produced by each strain was normalized on the base of the number of bacterial cells calculated in each test, as previously performed (Gabriele et al., 2022). The number of bacterial cells was estimated through the optical density of each liquid culture measured at 600 nm. The results are expressed as µg of IAA produced by 1.5 x 10^8^ bacterial cells, corresponding to the McFarland Standard n° 0.5 (Mc Farland, 1907).

### 2.8 Bacterial stress resistance assay

According to Li et al. (2021), the bacterial resistance pattern toward a panel of five different stresses was assessed for one representative strain for each detected haplotype, as performed by Gabriele et al. (2022). Isolated yeasts were not included in these analyses. The five conditions (Li et al., 2021) were 0.0025% H_2_O_2_ (oxidative stress), 15% polyethylene glycol (PEG)−6000 (water potential stress), 2% NaCl (salt stress), 1 µg/mL streptomycin and 5 µg/mL penicillin. According to Li et al. (2021), the two antibiotics were selected on the base of the antibiotics commonly produced by rhizosphere microorganisms as indicators of biotic stress resistance. The tests were conducted in 96-well microplates in triplicate for each tested substance, according to Chiellini et al. (2018), in Mueller Hinton Broth (MHB) and a total volume of 100 µL. Both a positive control (the bacterial inoculum in MHB medium) and a negative control (MHB medium) were set up. Bacterial growth was determined as optical density at 600 nm (OD600), after 48 h of growth at 27 °C without shaking. According to Chiellini et al. (2018), the OD600 value measured for the positive control was considered as 100% of the growth value; the other measured values were reported as the percentage of growth in proportion to the positive control. As each test was conducted in triplicate, the average value was considered for the results and discussion.

### 2.9 Preparation of plant extract

The *B. bituminosa* fresh aerial parts were extracted with chloroform (w:v, 1:10) by ultrasound-assisted extraction using a Labsonic LBS2 ultrasonic bath (52 Hz) for 1 h at 30° C and then kept under stirring for 24 h. The extraction process was repeated three times. The extract solutions were filtered through filter paper, combined, and then dried by a rotatory evaporator and kept at -20°C until analyses. The dried chloroformic extract was dissolved in methanol to obtain a solution with a concentration of 2 mg/mL for chemical and biological analyses.

### 2.10 Ultra-high performance liquid chromatography-high resolution orbitrap/mass spectrometry (UHPLC-HR-Orbitrap/MS)

The chemical analysis of the chloroform extract obtained from the aerial parts of *B. bituminosa* wild plant was performed using an ultra-high performance liquid chromatography (UHPLC, Vanquish Flex Binary pump) coupled with an electrospray ionization (ESI) source high-resolution mass spectrometer (HR-MS) Q Exactive Plus Orbitrap-based FT-MS system (Thermo Fischer Scientific Inc., Bremen, Germany).

The dried chloroform extract was dissolved in methanol (2 mg/mL), centrifuged at 4000 rpm for 10 min and 5 µL of the supernatant was injected in the UHPLC-MS system equipped with a C-18 Kinetex^®^ Biphenyl column (100 × 2.1 mm, 2.6 µm particle size) provided of a Security Guard TM Ultra Cartridge (Phenomenex, Bologna, Italy). The elution was performed with a mixture of methanol acidified with formic acid 0.1% (solvent A) and H_2_O acidified with formic acid 0.1% (solvent B) using a solvent gradient 30-85% A in 22 min, at a flow rate of 0.5 mL/min. Column and autosampler temperatures were maintained at 35 and 4°C, respectively. The acquisition of HR mass spectra was done in a scan range of *m/z* 135-2000 in ESI positive ionization mode, operating in full (70000 resolution, 220 ms maximum injection time) and data dependent-MS/MS scan (17500 resolution, 60 ms maximum injection time). Ionization parameters were optimized as previously reported (Pieracci et al., 2022). Data were elaborated using the Xcalibur™ software.

### 2.11 Disk-diffusion assay to test the antibacterial activity of the plant extract

The plant extract was tested against six bacterial strains (one representative strain for each detected haplotype) and against two bacterial strains present in the laboratory collection of the Institute of Agricultural Biology and Biotechnology of the National Research Council located in Pisa. These two strains were: *Pseudomonas stutzeri* DSM 5190 from the Leibniz Institute DSMZ-German Collection of Microorganisms and Cell Cultures (https://www.dsmz.de/ ) and the *Stenotrophomonas rhizophila* strain A, isolated from a Chlorella-like microalga and published in Serra et al. (2022) Isolated yeasts were not included in these analyses. The test was conducted to evaluate whether the plants might assert an effect in the control of its endophytic bacterial population. The agar disk diffusion assay based on the Kirby-Bauer test (Bauer et al., 1966) was performed by testing 10 µL of four different concentrations of the plant extract (0.2, 0.4, 0.8, and 2 mg/mL), together with 10 µg of streptomycin (control) and 10 µL of 96% ethanol (Merck). Each substance was dropped on sterile Whatman paper disks of 6 mm diameter and put on the surface of the agar plate inoculated with the overnight grown bacterial culture. The test was conducted on Mueller Hinton Agar (MHA) medium, in triplicate. Results were expressed as the average measure of the diameters of the halo surrounding each paper disk in the Petri dishes when the inhibition was present.

## 3 Results

### 3.1 Isolation of bacterial strains from plants grown *in vitro* and ARDRA screening for haplotype attribution

The growth of the bacterial colonies in the TSA plates made with serial dilutions, revealed that the lower dilutions exhibited lower bacterial load, while the higher dilutions, showed greater bacterial colonies both in the aerial parts and in the roots (data not shown). A total of 43 isolates were successfully recovered from the seeds and from the plants grown *in vitro*. Only one isolated bacterial strain was recovered from the seeds; 21 bacterial isolates were recovered from the plant aerial parts and 21 isolates from the roots (5 yeasts and 15 bacteria). ARDRA screening was performed on the 38 bacterial isolates, on the amplified 16S rRNA gene.

Results of the ARDRA screening (**Table 1**) revealed the presence of 6 different haplotypes among the bacterial endophytes. The 21 bacteria isolated from the aerial parts were divided into 3 haplotypes, two of which were co-dominant, hosting 10 strains each. The 16 bacteria isolated from the roots were distributed in 5 different haplotypes, one (haplotype B) was dominant with 8 isolates. The only strain isolated from the seed showed a different haplotype (haplotype E), not shared by the other bacteria isolated in the plant compartments.

**Table 1:** ARDRA screening results on the 38 bacterial isolated strains.

Haplotype ID	N° of bacteria from the aerial part*s*	N° of bacteria from the roots	N° of bacteria from the seeds	Total bacterial isolates
A	10	3	0	13
B	0	8	0	8
C	10	3	0	13
D	1	1	0	2
E	0	1	0	1
F	0	0	1	1
Total bacterial isolates	21	16	1	38

Considering that in the seeds only one bacterium was retrieved and cultivated, the diversity indices calculated on the base of the ARDRA haplotypes distribution (**Table 2**) highlighted the highest Shannon and Chao-1 values in roots (1.321 and 6, respectively), and the highest Evenness index value in the aerial part of the plants (0.7811).

**Table 2:** Diversity indices (Chao-1, Shannon and Evenness) calculated on the haplotypes obtained from ARDRA screening for each plant compartment.

	Aerial part	Roots	Seeds
Shannon_H	0.8516	1.321	0
Evenness_e^H/S	0.7811	0.7493	1
Chao-1	3	6	1

### 3.2 Molecular identification of isolated bacterial strains

**Figure 1:**
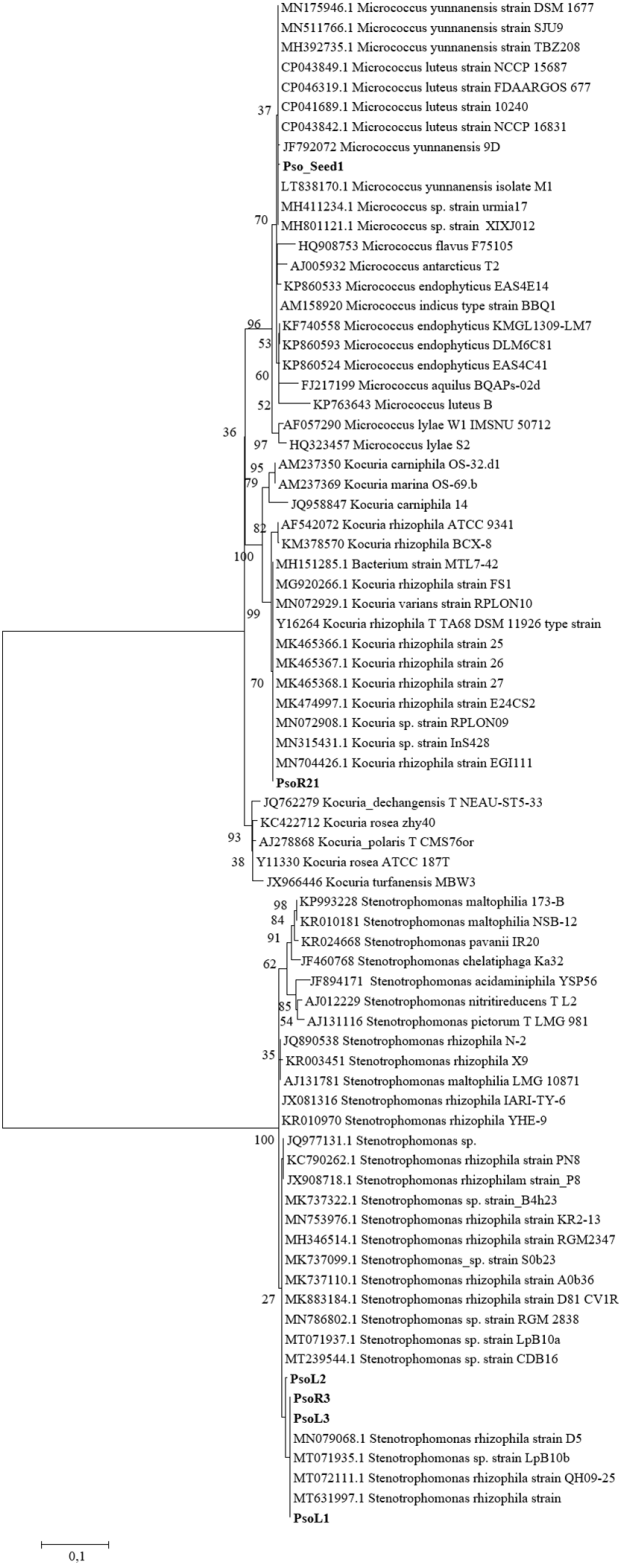
Phylogenetic tree reconstruction based on the 16S rRNA gene, obtained with maximum likelihood method on a total of 77 sequences, 6 of them belonging to our strains (highlighted in bold) and 71 high quality sequences selected among those most similar to our isolated bacteria.

One representative strain for each haplotype was taxonomically identified through amplification and sequencing of the 16S rRNA gene (**Table 3**). Results highlighted that the majority of our isolated strains is closely related to the species *Stenotrophomonas rhizophila*. These strains are those grouped into the haplotypes A, B, C, and D. Interestingly, the first described species that are more like our strains, are not the same in the four isolates, and it is possible to individuate three different *Stenotrophomonas rhizophila* close relatives (Acc. Nrs MT631997.1, KC790262.1, and MN753976.1). Strain Pso_R21 showed a 99.9% similarity with *Kocuria rhizophila*, and the only strain isolated from seeds is phylogenetic related to *Micrococcus luteus*. The phylogenetic analysis of these sequences (**Figure 1**) revealed that the four sequences related to *S. rhizophila* and representative of 95.3% of the total isolated strains (41 strains of 43 total), cluster together within the clade of *Stenotrophomonas rhizophila*. The analysis evidenced a high similarity between strains Pso_L1, Pso_L3, and Pso_R3 (haplotypes B, C, and D), and a slight separation of strain Pso_L2 (haplotype A) showing basal position concerning the previous three strains in the tree topology. According to BLAST analysis, strain Pso_R21 is part of the *Kocuria* sp. clade, closely related with *K. rhizophila*. On the other side, despite strain Pso_Seed_1 falling within the clade of *Micrococcus* sp., it seems very closely related to *Micrococcus yunnanensis* (**Figure 1**).

**Table 3:** Taxonomic affiliation of the bacterial isolates according to BLAST analysis.

Isolate	Haplotype	Length (bp)	Accession number	Similarity 1 blast	Similarity first described
Pso_L1	D	870	OP389135		MT631997.1 *Stenotrophomonas rhizophila* strain LA-3 16S 100%
Pso_L2	A	1139	OP389136		KC790262.1 *Stenotrophomonas rhizophila* strain PN8 99.65%
Pso_L3	C	1056	OP389137	MT239544.1 *Stenotrophomonas* sp. 99.91%	MN753976.1 *Stenotrophomonas rhizophila* strain KR2-13 99.91%
Pso_R3	B	1083	OP389138		KC790262.1 *Stenotrophomonas rhizophila* strain PN8 100%
Pso_R21	E	1024	OP389139		MN704426.1 *Kocuria rhizophila* strain EGI111 99.9%
Pso_Seed_1	F	1169	OP389140		CP041689.1 *Micrococcus luteus* strain 10240 99.4%

### 3.3 Phenotypic characterization of the bacterial isolates

#### 3.3.1 Antagonism among endophytes and biosurfactant production assay

The antagonism assay through the cross-streaking method was performed on both bacterial and yeast isolates. Results are shown in **Supplementary Table S1** and highlight that there is not any antagonistic effect asserted by the isolates from roots and seeds, nor against those isolated from the aerial part of the plants and the seeds. Accordingly, all the target strains (aerial parts and seeds) were able to grow in presence of each tester strain (roots and seeds), after 48 h. An example of the executed test is reported in **Supplementary Figure S1**, where it is possible to observe that the growth of the strains in the test is analogous to that of the control plates (**Supplementary Figure S2**).

In addition, the biosurfactant production revealed that any of the isolated strains were able to produce biosurfactants since no blue color appeared in the agar plates with the Mineral Salts Agar medium. Accordingly, only the five isolated yeasts were able to grow in such a medium, without producing any biosurfactant molecule (**Supplementary Figure S3**).

#### 3.3.2 IAA production assay

The IAA production assay performed on all the isolates, expressed as µg IAA in 1.5 x 10^8^ bacterial cells, is reported in **Table 4**. Results revealed that isolates Pso_L7, Pso_L16, and Pso_L19 from the aerial part, as well as strains Pso_R11 and Pso_R12 from the roots and Seed_Pso1 from the seeds, were able to produce the highest amount of IAA, higher than 1 µg for 10^8^ cells. The lowest values of the produced IAA were measured for a strain isolated from the roots, Pso_R21 accounting for less than 0.1 µg IAA for 10^8^ cells. Overall, among the strains isolated from the aerial part of the plants, 11 were able to produce an amount of IAA for 10^8^ cells greater than 0.6 µg (Pso_L2, L4, L5, L7, L8, L12, L13, L15, L16, L19, L21, corresponding to more than 50% of the isolates from this plant compartment), while only 3 strains isolated from the roots were able to produce the same amount of IAA (Pso_R8, R11, R12, corresponding to about 18.75% of the bacterial roots isolates). Also, the single isolated strain from the seeds (Pso_Seed_1) was included among those strains with a high IAA production.

**Table 4:** Haplotype attribution, taxonomy, plant source, and IAA production of the endophytic bacterial strains isolated from the plants.

*Strain*	*Plant source*	*Organism*	*µg IAA/1.5 x 10^8^ cells*	*ARDRA haplotype - 16S*	*Taxonomic affiliation*
*Pso_L1*	Aerial part	Bacterium	0.14	D	*Stenotrophomonas rhizophila*
*Pso_L2*	Aerial part	Bacterium	0.65	A	*Stenotrophomonas rhizophila*
*Pso_L3*	Aerial part	Bacterium	0.36	C	*Stenotrophomonas rhizophila*
*Pso_L4*	Aerial part	Bacterium	0.69	A	*Stenotrophomonas rhizophila*
*Pso_L5*	Aerial part	Bacterium	0.77	C	*Stenotrophomonas rhizophila*
*Pso_L6*	Aerial part	Bacterium	0.28	C	*Stenotrophomonas rhizophila*
*Pso_L7*	Aerial part	Bacterium	1.02	C	*Stenotrophomonas rhizophila*
*Pso_L8*	Aerial part	Bacterium	0.77	A	*Stenotrophomonas rhizophila*
*Pso_L9*	Aerial part	Bacterium	0.35	C	*Stenotrophomonas rhizophila*
*Pso_L10*	Aerial part	Bacterium	0.59	C	*Stenotrophomonas rhizophila*
*Pso_L11*	Aerial part	Bacterium	0.36	A	*Stenotrophomonas rhizophila*
*Pso_L12*	Aerial part	Bacterium	0.72	C	*Stenotrophomonas rhizophila*
*Pso_L13*	Aerial part	Bacterium	0.63	A	*Stenotrophomonas rhizophila*
*Pso_L14*	Aerial part	Bacterium	0.41	C	*Stenotrophomonas rhizophila*
*Pso_L15*	Aerial part	Bacterium	0.77	A	*Stenotrophomonas rhizophila*
*Pso_L16*	Aerial part	Bacterium	1.31	A	*Stenotrophomonas rhizophila*
*Pso_L17*	Aerial part	Bacterium	0.36	A	*Stenotrophomonas rhizophila*
*Pso_L18*	Aerial part	Bacterium	0.47	A	*Stenotrophomonas rhizophila*
*Pso_L19*	Aerial part	Bacterium	1.47	C	*Stenotrophomonas rhizophila*
*Pso_L20*	Aerial part	Bacterium	0.27	A	*Stenotrophomonas rhizophila*
*Pso_L21*	Aerial part	Bacterium	0.63	C	*Stenotrophomonas rhizophila*
*Pso_R1*	Roots	Bacterium	0.33	C	*Stenotrophomonas rhizophila*
*Pso_R2*	Roots	Bacterium	0.43	A	*Stenotrophomonas rhizophila*
*Pso_R3*	Roots	Bacterium	0.43	B	*Stenotrophomonas rhizophila*
*Pso_R4*	Roots	Bacterium	0.39	B	*Stenotrophomonas rhizophila*
*Pso_R5*	Roots	Bacterium	0.37	B	*Stenotrophomonas rhizophila*
*Pso_R6*	Roots	Bacterium	0.54	B	*Stenotrophomonas rhizophila*
*Pso_R7*	Roots	Bacterium	0.43	B	*Stenotrophomonas rhizophila*
*Pso_R8*	Roots	Bacterium	0.91	A	*Stenotrophomonas rhizophila*
*Pso_R9*	Roots	Bacterium	0.28	C	*Stenotrophomonas rhizophila*
*Pso_R10*	Roots	Bacterium	0.55	C	*Stenotrophomonas rhizophila*
*Pso_R11*	Roots	Bacterium	1.02	A	*Stenotrophomonas rhizophila*
*Pso_R12*	Roots	Bacterium	1.01	D	*Stenotrophomonas rhizophila*
*Pso_R13*	Roots	Bacterium	0.35	B	*Stenotrophomonas rhizophila*
*Pso_R14*	Roots	Bacterium	0.55	B	*Stenotrophomonas rhizophila*
*Pso_R16*	Roots	Bacterium	0.54	B	*Stenotrophomonas rhizophila*
*Pso_R21*	Roots	Bacterium	0.03	E	*Kocuria rhizophila*
*Pso_Seed_1*	Seeds	Bacterium	1.31	F	*Micrococcus luteus*

Yeast strains Pso_R15, 17, 18, 19, and 20 are removed from the analysis.

### 3.4 Stress tolerance test and susceptibility to the plant extract

The tolerance stress against five different conditions (**Table 5**) revealed three distinct patterns. Strains Pso_L1, Pso_L2, Pso_L3, and Pso_R3, representatives of haplotypes A, B, C, and D, showed a complete tolerance against both the tested antibiotics, weak growth in the presence of NaCl and PEG 6000, and high sensitivity in presence of oxidative stress. Strain Pso_R21, the only representative of haplotype E, showed resistance only in presence of streptomycin, and a slightly reduced growth in presence of PEG 6000, together with a moderate growth in presence of the three remaining stresses. Finally, the only strain isolated from seeds and representative of haplotype F showed resistance only in presence of streptomycin and a total sensitivity in presence of all the other four conditions.

**Table 5:** Stress tolerance patterns against 5 µg/mL penicillin, 1 µg/mL streptomycin, 2% NaCl, 15% PEG−6000, and 0.0025% H_2_O_2_, for the six strains representing the six detected haplotypes.

	Haplotype	Pen 5 μg/mL	Strept 1 μg/mL	NaCl 2%	PEG 6000 15%	H_2_O_2_ 0.0025%
Pso_L1	D					
Pso_L2	A					
Pso_L3	C					
Pso_R3	B					
Pso_R21	E					
Pso_Seed_1	F					

Growth values below 10% are indicated with red colour and correspond to “absence of growth”; growth values between 10 and 50% are evidenced in orange colour, and correspond to “weak growth”; values between 50 and 75% indicated in yellow, correspond to “growth”, and values greater than 75% (green colour) correspond to “complete growth”.

The agar disk diffusion assay conducted on the six strains representative of each haplotype revealed that the solvent used for the dilution of the plant extract (ethanol 96%) does not have any effect on the strain growth/survival (**Table 6**). All the strains except for the one isolated from the seeds are sensitive to streptomycin (10 µg), that in this test was used at a higher concentration with respect to the stress tolerance test, in which they were all resistant. Strain Pso_L3 representative of haplotype C is the most sensitive to the plant extract, even at the lowest tested concentration. Strain Pso_R3 is slightly sensitive to the highest concentration (2 mg/mL of plant extract) while all the other strains are not sensitive. *S. rhizophila* strain A isolated from the phycosphere of a microalga, as well as the *P. stutzeri* strain DSM 5190, are not sensitive to the tested concentration of the plant extract.

**Table 6:** The measure of the halo diameter (mm) produced by the lack of growth of the bacterial strains in the presence of different concentrations of the tested plant extract.

				Plant extract concentration (mg/mL)			
	Haplotype	Streptomycin	EtOH 96%	0.2	0.4	0.8	2
**Pso_L_1**	D	12	0	0	0	0	0
**Pso_L_2**	A	11	0	0	0	0	0
**Pso_L_3**	C	11	0	8	8	10	9
**Pso_R_3**	B	11	0	0	0	0	8
**Pso_R_21**	E	21	0	0	0	0	0
**Pso_Seed_1**	F	0	0	0	0	0	0
**A_*S. rhizophila***	–	9	0	0	0	0	0
**DSM 5190**	–	11	0	0	0	0	0

Streptomycin was tested as the control antibiotic and EtOH 96% was tested as it was used as a solvent for the plant extract.

### 3.5 Chemical characterization of *B. bituminosa* chloroform extract

**Figure 2:**
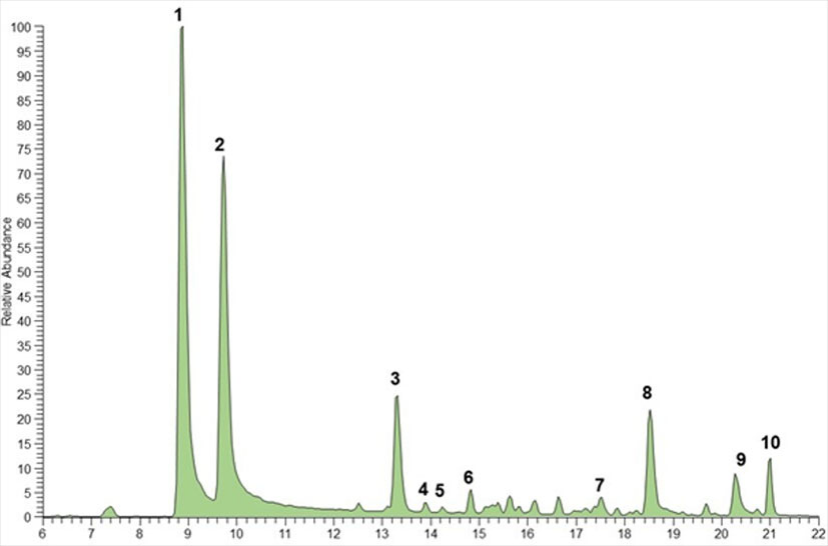
UHPLC-HR-ESI- Orbitrap/MS chromatogram (positive ionization mode) of (*B*) *bituminosa* chloroform extract wild plant. Peak data are shown in **Table 7**.

The chemical composition of the chloroform extract of *B. bituminosa* wild plant was established through UHPLC-HR-ESI-MS/Orbitrap analysis. The chromatogram obtained operating in positive ionization mode is reported in **Figure 2**. All compounds were identified based on their elution order, full and fragmentation MS data (**Table 7**) compared with the literature data, considering an accepted mass error <5 ppm. The extract was characterized by four main chemical classes of compounds; furanocoumarins were the most represented, with the two isomers psoralen (peak 1) and angelicin (peak 2) detected as the main components ([M+H]^+^ at *m/z* 187.0389), according to previous studies demonstrating their occurrence in *B. bituminosa* (Innocenti et al., 1997; Pistelli et al., 2017), as well as in *Psoralea* genus (Zhao et al., 2005). Proceeding in the elution order, the hydroxycinnamic acid plicatin B (peak 3, [M+H]^+^ at *m/z* 247.1327) was tentatively identified, as it was previously reported as *B. bituminosa* component by Pistelli et al. (2003). Peaks 4 and 5, showing the same protonated molecular ion [M+H]^+^ at *m/z* 325.1431, were two prenylated flavanone isomers commonly found in *Psoralea* genus, tentatively identified as (iso)bavachin and (iso)bavachalcon, respectively, based on mass fragmentation pathways (Zhao et al., 2005). Two additional prenylated flavanones (peak 6, [M+H]^+^ at *m/z* 339.1225; peak 7, [M+H]^+^ at *m/z* 339.1588), were identified as corylifol C and bavachinin, a methylated form of bavachin, respectively (Zhao et al., 2005). To the best of our knowledge, prenylated flavanones were herein detected for the first time in *B. bituminosa*. Finally, as expected based on literature evidence (Pistelli et al., 2003), prenylated pterocarpans were detected and identified as erybraedin C (peak 8, [M+H]^+^ at *m/z* 393.2055) and bitucarpin A (peak 9, [M+H]^+^ at *m/z* 353.1743). Peak 10 and other minor peaks remained unidentified.

**Table 7:** Chromatographic and mass spectrometry data of compounds tentatively identified in *B. bituminosa* chloroform extract by UHPLC-HR-Orbitrap/MS.

Peak	Compound^a^	*t*_R_ (min)	[M+H]^+^	MS/MS ions (*m/z*)^b^	Molecular Formula	Mass Error (ppm)
Furanocoumarins						
**1**	Psoralen	8.9	187.0389	115.05, **131.05**, 143.05	C_11_H_6_O_3_	-0.64
**2**	Angelicin (isopsoralen)	9.7	187.0389	115.05, **131.05**, 143.05, 159.04	C_11_H_6_O_3_	-0.43
Hydroxycinnamic acid						
**3**	Plicatin B	13.3	247.1327	69.07, **191.07**, 215.11	C_15_H_18_O_3_	-0.53
Prenylated flavanones						
**4**	(Iso)bavachin	14.1	325.1431	269.08, 191.11, 135.04, **123.04**	C_20_H_20_O_4_	-0.92
**5**	(Iso)bavacalchone	14.2	325.1431	269.08, **191.11**, 149.06, 123.04	C_20_H_20_O_4_	-0.92
**6**	Corylifol C	14.8	339.1225	283.06, 255.07,	C_20_H_18_O_5_	-0.59
**7**	Bavachinin	17.5	339.1588	283.10, 191.11, **137.06**	C_21_H_22_O_4_	-0.88
Pterocarpans						
**8**	Erybraedin C	18.5	393.2055	69.00, 135.04, **191.11**	C_25_H_28_O_4_	-1.27
**9**	Bitucarpin A	20.3	353.1743	137.06, **149.06**, 205.12	C_22_H_24_O_4_	-1.36
Unknown						
**10**	Unidentified	21.0	371.3153	101.06, 129.05, 147.07	−	−

a Compound numbers correspond to those in **Figure 2**. ^b^The base ion peak is indicated in bold.

## 4 Discussion

Endophytes include beneficial microorganisms exerting well-known growth-promoting activities in plants, such as seed germination, growth support, nutrient supply, stimulation in metabolite production, and resistance against biotic and abiotic stress factors (Berg, 2014). Bacterial endophytes are most often located within the plant’s intercellular spaces, which are rich in carbohydrates, amino acids, and inorganic nutrients; they are present in all the plant tissues, including roots, leaves, stems, flowers, and also in seeds (Taulé et al., 2021). Interestingly, endophytes can affect the plant phenotype determining its phytochemical profile and mediating the expression of the plant functional traits too (Harrison and Griffin, 2020).

In this work, we isolated and characterized for the first time the bacterial endophytes from seeds and from the tissues of 40-day plants of *B. bituminosa* grown *in vitro*. Endophytes isolated from both roots and aerial parts showed differences in the composition and the phenotypic traits of the isolated strains. The highest values of Shannon and Chao-1 diversity indices calculated on the basis of the haplotype distribution were retrieved in roots; the highest Evenness index value was highlighted in the aerial parts of the plants. According to the definition of the Shannon index, the higher value observed in the roots compartment (1.321) with respect to the aerial part (0.8515) and the seeds (0), suggested a higher genetic diversity of the bacterial isolates in this compartment. A similar observation was evidenced by the analysis of the Chao-1 index, giving indications about the species richness in a sample, which showed higher values for the root community (6) rather than for the aerial parts (3) and the seeds (1). This observation was in agreement with many previous works (Chiellini et al., 2014), showing a greater culturable bacterial diversity in root endophytes compared to the leave community.

It is worth noticing that only one bacterial endophyte closely related to the species *Micrococcus* was isolated from the seeds. All other strains isolated from both roots and aerial parts were not retrieved in seeds. This aspect might find different explanations from the available literature. The cultivation of seed endophytes is challenging because of the specific habitat of origin and a large fraction of the bacterial endophytic population in seeds probably has unknown cultivation conditions (Truyens et al., 2015). Moreover, the culturable seed microbial communities are considered to be limited in their size (Mundt and Hinkle, 1976; Shade et al., 2017) and many strains of the seed microbiota may be inactive or dormant (Vriezen et al., 2012; Jiang et al., 2016), or are in a viable but not-culturable state (Truyens et al., 2015). Therefore, we cannot exclude that the bacterial strains found in roots and aerial parts might have not been retrieved in the seeds for these reasons. Bacterial plate counts revealed that the highest number of bacterial colonies were retrieved in the most diluted samples and at the same time the lowest counts were detected in the most concentrated samples(Data not shown). This result might suggest the presence of some molecules produced by the plant, which might assert a kind of “control” on the endophytic population, acting on the viability and number of individuals.

The aerial parts of wild *B. bituminosa* plants have been extensively examined either for the characterization of their peculiar bitumen smell (Bertoli et al., 2004; Tava et al., 2007) and other phytochemicals (Pistelli et al., 2003; Pecetti et al., 2016; Pistelli et al., 2017; Ramli et al., 2022). The plant extract, herein examined by the UHPLC-HR-ESI-Orbitrap/MS, showed the presence of secondary metabolites previously recognized in other samples Pistelli et al., 2017). The furanocoumarins psoralen and angelicin, representing the main chemical compounds found in the analysed extract, were reported by Baig (2022) to have significant antibacterial activity against different gram-positive and gram-negative bacteria. The effectiveness of different prenylated flavonoids, isolated from *Psoralea corylifolia* L., on *Escherichia coli* and *Staphylococcus aureus* was also investigated. Among these molecules, bavachalcone and isobavacalchone have been shown to be more effective on the gram-positive bacteria (*S. aureus*) than on the gram-negative ones (*E. coli*). However, for both bacterial strains, these compounds showed a good IC_50_ value, better than the furanocoumarins previously mentioned (Baig, 2022). Likewise, Yin et al. (2004) evidenced remarkable inhibitory properties on *S. aureus* and *S. epidermidis* of some of the prenylated flavonoids found in the present extract, especially bavachalcone, isobavachalcone, bavachin, and bavachinin. Prenylated flavonoids have recently gained increasing attention due to their activity against different bacteria strains, showing good to strong antibacterial power (Pistelli and Giorgi, 2012). Their activity was strictly connected to their chemical structure; indeed, as reported by Pistelli and Giorgi (2012), the presence of a prenyl group at the A-ring in the chalcone derivatives made the compounds active, as in the case of isobavachalcone. On the contrary, if the prenyl group at A-ring is oxygenated or/and further cyclized, the compounds become inactive, while the presence of a 7-methoxy group makes a molecule more active than the analogs with a 7-hydroxy group (Pistelli and Giorgi, 2012).

Other compounds found in the aerial parts were the pterocarpans, known as biologically active isoflavonoids able to work as phytoalexins, antimicrobial molecules with the ability in plant defense against pathogens (Selvam et al., 2017). However, little has been reported in the literature concerning the antibacterial properties of erybraedin C and bitucarpin A. All the secondary metabolites could contribute to the determination of a specific relationship with the different bacterial haplotypes. Noteworthily, the distribution of metabolites during plant development can be different (Bertoli et al., 2004; Pistelli et al., 2017).

Interestingly, results on the sensitivity of isolated endophytes to the plant extract revealed that two strains, Pso_L3 and Pso_R3 representing haplotypes C and B, respectively, are the most sensitive to the tested plant extract. In particular, the strain Pso_L3 (haplotype C) is sensitive to all the tested concentrations and Pso_R3 (haplotype B) to the highest one. It is worth noticing that haplotype C is composed of most of the strains isolated from the aerial part (10 out of 13 total isolates) while, haplotype B is composed only of strains isolated from the roots. This result might suggest a differential sensitivity of our strains to different compounds, thus lead to the presence of a kind of control exerted by the plant in the endophytic population and its distribution at the very early stages of development. In light of this hypothesis, future analysis will be addressed to test the pure compounds extracted from the plants for their inhibitory effect against the isolated bacterial endophytes.

One of the factors affecting the distribution of endophytic bacterial communities within the plant tissues might be the antagonistic interactions among strains (Maida et al., 2016; Maggini et al., 2018). This observation was not valid for our 40-day plants of *B. bituminosa*, whose endophytic community did not show any antagonism among strains. However, we have to consider that our analysis was performed at the earliest stages of the plant growth, differently from the previously cited works (Maida et al., 2016; Maggini et al., 2018). Accordingly, we cannot exclude that the shaping of the endophytic community within the *B. bituminosa* plant tissues due to antagonistic interactions among strains, might occur at the later stages of the plant development. Indeed, during its life and growth, the plant selects the endophytic community from different “sources” (Frank et al., 2017). Soil represents the first initial inoculum of endophytic microorganisms for plants and the native soil composition is considered important for endophyte recruitment (Compant et al., 2016). At the rhizospheric level, plants release significant amounts of substances, especially through root exudates, influencing the rhizospheric microbial communities. Our research was conducted using *in vitro* plants, so we cannot exclude that most of the interactions among endophytes might occur in the late stages of plant development, at least when the endophytic bacterial community is already selected by the plant.

Antagonism is not the only factor shaping endophytic communities within plant tissues because of the localization of bacterial endophytes in the intercellular spaces (Taulé et al., 2021). Bacterial endophytes are most often located within the plant intercellular spaces but can be found in all plant tissues (Taulé et al., 2021). Consequently, also the plant genotype has a central role in endophyte selection/colonization (Kandel et al., 2017). The presence of different kinds and different amounts of substances produced by the plant in its tissues, might have a role in the shaping of the endophytic bacterial community too. According to the literature, several investigations were performed to characterize the metabolomic profile of *B. bituminosa* aerial parts. The volatile profile has been determined as a pool of compounds with peculiar behavior depending on organs and developmental phase (Bertoli et al., 2004; Tava et al., 2007). Also, proteins and sugars were determined in adult plants because of their contribution to the nutritional value of *B. bituminosa* aerial parts (Ventura et al., 2000).

The phenotypic characterization of the bacterial strains revealed different characteristics and peculiar patterns of each strain that was not always related to the taxonomy, nor the plant compartment of “origin”. The IAA quantification revealed a higher production from the endophytes isolated from the aerial parts and a lower production from those of the roots. Interestingly, the only isolated strain from the seed is among those producing the highest IAA amount. According to our data, the IAA production ability in the endophytes seems to be somehow related to the plant compartment.

The tolerance stress against five different conditions revealed three distinct patterns. Strains Pso_L_1, Pso_L_2, Pso_L_3, and Pso_R_3, representatives of haplotypes A, B, C, and D, showed complete tolerance against both tested antibiotic, weak growth in presence of NaCl and PEG 6000, and high sensitivity in presence of oxidative stress. Interestingly, these three strains belong to the same bacterial species, even though ARDRA screening attributed them to three different haplotypes. The strain isolated from the seeds and strain Pso_R_21 have their peculiar tolerance pattern. These two strains were also taxonomically related to different bacterial species. The strain isolated from the seeds showed the lowest resistance toward the tested environmental stresses. Overall, stress analysis patterns revealed that the differences among strains seem to be mainly related to taxonomy and the plant compartment.

Our data did not reveal any ability of our isolated strains in producing biosurfactants. Despite these results, we cannot exclude any ability to produce biosurfactant molecules different from that specifically detected by the test that we chose in our research. Indeed, the Mineral Salts Agar medium method test is specific for anionic biosurfactants and, in particular, it was developed to detect rhamnolipids.

## 5 Conclusions

The bacterial culturable endophytic community was isolated from different organs of *in vitro* plants at 40-day growth, for the first time. Bacterial strains were identified and characterized for different phenotypic traits, such as the IAA and biosurfactants production ability, the resistance to different biotic and abiotic stresses, the antagonistic ability among each other. Interestingly, strains were exposed to different concentrations of *B. bituminosa* plant extract showing different susceptibility, probably determined by different secondary metabolites produced by the plant and depending on the isolation source (aerial parts and roots). Bacterial strains were subdivided into 6 haplotypes, dominated by a single species related to *Stenotrophomonas rhizophila* and showing different phenotypic characteristics. The obtained results open new perspectives for future analysis addressed to test the sensitivity of bacterial endophytes towards the pure compounds extracted from *B. bituminosa*, and to investigate the role of these compounds on the distribution of endophytes within the different plant tissues.

## Data availability statement

The datasets presented in this study can be found in online repositories. The names of the repository/repositories and accession number(s) can be found below:https://www.ncbi.nlm.nih.gov/, OP389135; https://www.ncbi.nlm.nih.gov/, OP389136; https://www.ncbi.nlm.nih.gov/ , OP389137; https://www.ncbi.nlm.nih.gov/, OP389138; https://www.ncbi.nlm.nih.gov/, OP389139; https://www.ncbi.nlm.nih.gov/, OP389140.

## Author contributions

LP, CC, and MDL conceived and designed the experiments; LP, CC, MDL, and YP performed the experiments; LP, CC, and MDL analysed the data; LP, MDL, and VL supervised the project; LP, CC, MDL, and YP prepared the draft. All authors have read and agreed to the published version of the manuscript
