# Supplementary material for: Characterization of the endophytic bacterial community of Bituminaria bituminosa plant grown in vitro and its interaction with the plant extract
Source: Front Plant Sci. 2023 Jan 18;13:1076573. doi: 10.3389/fpls.2022.1076573 (PMC9889976; doi:10.3389/fpls.2022.1076573)
Supplement: Supplementary file 1 [file DataSheet_1.pdf]

SUPPLEMENTARY MATERIAL

Characterization of the endophytic bacterial community of *Bituminaria bituminosa* plant grown *in vitro* and its interaction with plant extract

Carolina Chiellini[a], Marinella De Leo[b,c*], Vincenzo Longo[a], Ylenia Pieracci[b], Laura Pistelli[d,c*]

[a] Institute of Agricultural Biology and Biotechnology, Italian National Research Council, Via Moruzzi, 1, 56124 Pisa (Italy)

[b] Department of Pharmacy, University of Pisa, Via Bonanno Pisano 33, 56126 Pisa (Italy)

[c] Center for Instrument Sharing of Pisa, University (CISUP), Lungarno Pacinotti 43, 56126 Pisa, Italy

[d] Department of Agriculture, Food and Environment, University of Pisa, Via del Borghetto 80, 56124 Pisa (Italy)

*Corresponding authors: marinella.deleo@unipi.it; laura.pistelli@unipi.it

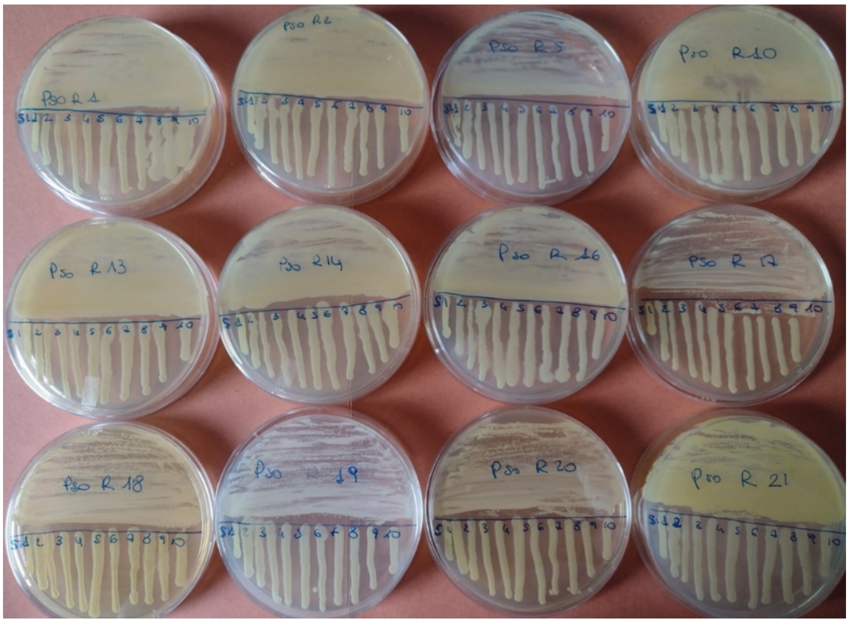

Supplementary Figure S1: example of Cross-streaking test performed on a part of the isolated bacterial and yeast strains.

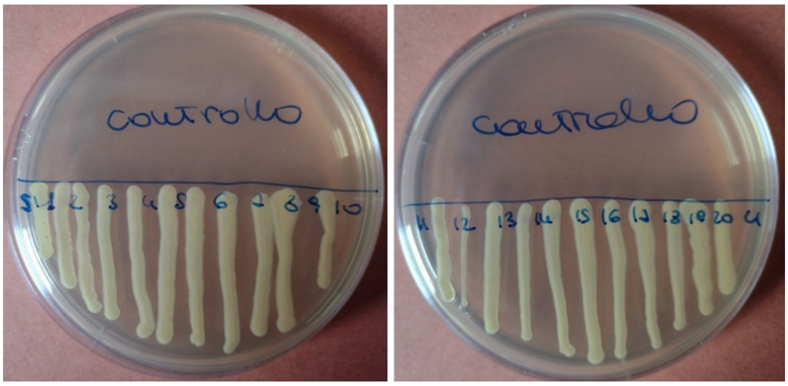

Supplementary Figure S2: Control plates for the Cross-streaking tests on all the isolated bacterial and yeast strains.

Supplementary Table S1: result of the cross streaking experiment performed between the seed and root isolated strains (tester strains) and the seed and aerial part isolated strains (target strains).

		Tester strains - Roots and Seeds																					
		Pso_R_1	Pso_R_2	Pso_R_3	Pso_R_4	Pso_R_5	Pso_R_6	Pso_R_7	Pso_R_8	Pso_R_9	Pso_R_10	Pso_R_11	Pso_R_12	Pso_R_13	Pso_R_14	Pso_R_15	Pso_R_16	Pso_R_17	Pso_R_18	Pso_R_19	Pso_R_20	Pso_R_21	ES_1_Pso
Target strains - leaves and seeds	Control	+	+	+	+	+	+	+	+	+	+	+	+	+	+	+	+	+	+	+	+	+	+
	ES_1_Pso	+	+	+	+	+	+	+	+	+	+	+	+	+	+	+	+	+	+	+	+	+	+
	Pso_L_1	+	+	+	+	+	+	+	+	+	+	+	+	+	+	+	+	+	+	+	+	+	+
	Pso_L_2	+	+	+	+	+	+	+	+	+	+	+	+	+	+	+	+	+	+	+	+	+	+
	Pso_L_3	+	+	+	+	+	+	+	+	+	+	+	+	+	+	+	+	+	+	+	+	+	+
	Pso_L_4	+	+	+	+	+	+	+	+	+	+	+	+	+	+	+	+	+	+	+	+	+	+
	Pso_L_5	+	+	+	+	+	+	+	+	+	+	+	+	+	+	+	+	+	+	+	+	+	+
	Pso_L_6	+	+	+	+	+	+	+	+	+	+	+	+	+	+	+	+	+	+	+	+	+	+
	Pso_L_7	+	+	+	+	+	+	+	+	+	+	+	+	+	+	+	+	+	+	+	+	+	+
	Pso_L_8	+	+	+	+	+	+	+	+	+	+	+	+	+	+	+	+	+	+	+	+	+	+
	Pso_L_9	+	+	+	+	+	+	+	+	+	+	+	+	+	+	+	+	+	+	+	+	+	+
	Pso_L_10	+	+	+	+	+	+	+	+	+	+	+	+	+	+	+	+	+	+	+	+	+	+
	Pso_L_11	+	+	+	+	+	+	+	+	+	+	+	+	+	+	+	+	+	+	+	+	+	+
	Pso_L_12	+	+	+	+	+	+	+	+	+	+	+	+	+	+	+	+	+	+	+	+	+	+
	Pso_L_13	+	+	+	+	+	+	+	+	+	+	+	+	+	+	+	+	+	+	+	+	+	+
	Pso_L_14	+	+	+	+	+	+	+	+	+	+	+	+	+	+	+	+	+	+	+	+	+	+
	Pso_L_15	+	+	+	+	+	+	+	+	+	+	+	+	+	+	+	+	+	+	+	+	+	+
	Pso_L_16	+	+	+	+	+	+	+	+	+	+	+	+	+	+	+	+	+	+	+	+	+	+
	Pso_L_17	+	+	+	+	+	+	+	+	+	+	+	+	+	+	+	+	+	+	+	+	+	+
	Pso_L_18	+	+	+	+	+	+	+	+	+	+	+	+	+	+	+	+	+	+	+	+	+	+
	Pso_L_19	+	+	+	+	+	+	+	+	+	+	+	+	+	+	+	+	+	+	+	+	+	+
	Pso_L_20	+	+	+	+	+	+	+	+	+	+	+	+	+	+	+	+	+	+	+	+	+	+
	Pso_L_21	+	+	+	+	+	+	+	+	+	+	+	+	+	+	+	+	+	+	+	+	+	+

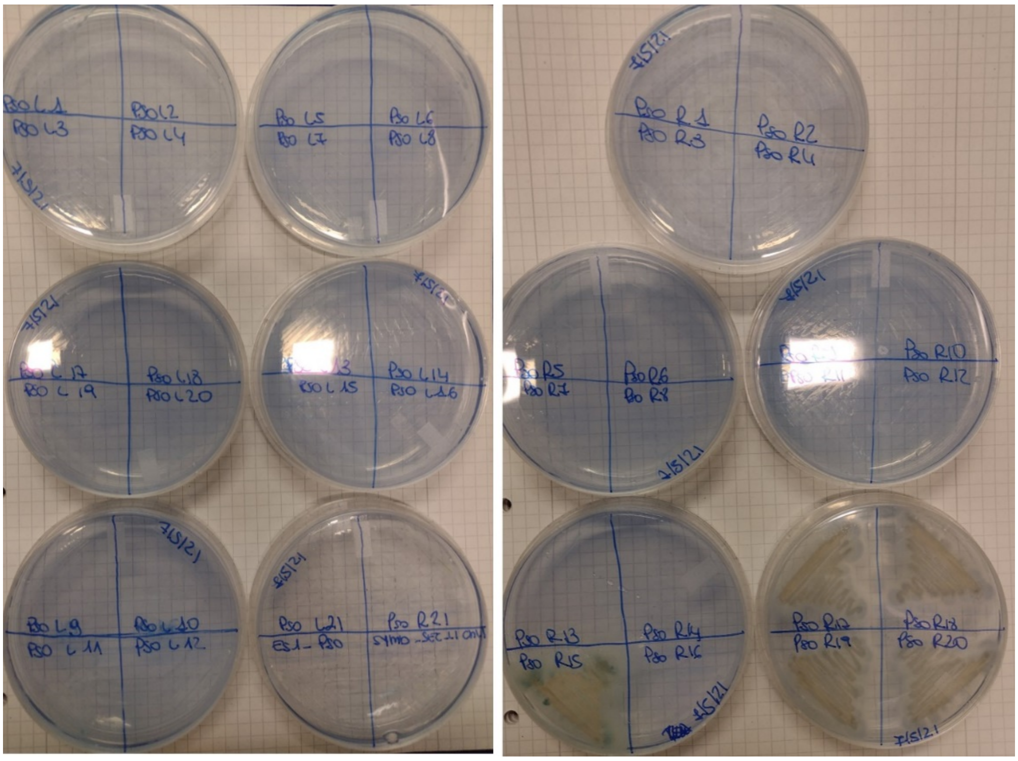

Supplementary Figure S3: example of biosurfactant production test on the isolated endophytic strains.
